# Comparative validity of the ASSO–Food Frequency Questionnaire for the web-based assessment of food and nutrients intake in adolescents

**DOI:** 10.3402/fnr.v59.26216

**Published:** 2015-04-15

**Authors:** Garden Tabacchi, Anna Rita Filippi, João Breda, Laura Censi, Emanuele Amodio, Giuseppe Napoli, Antonino Bianco, Monèm Jemni, Alberto Firenze, Caterina Mammina

**Affiliations:** 1 Department of Sciences for Health Promotion and Mother Child Care ‘G. D’Alessandro’, University of Palermo, Palermo, Italy; 2 Division of Non-communicable Diseases and Life-Course, World Health Organization Regional Office for Europe, Copenhagen, Denmark; 3 Agricultural Research Council, Food and Nutrition Research Centre (CRA-NUT), Rome, Italy; 4 Sport and Exercise Sciences Unit, University of Palermo, Palermo, Italy; 5 Centre for Sport and Human Performance, University of Greenwich, London, UK

**Keywords:** food frequency questionnaire, intake, nutrient, adolescent, validation, validity

## Abstract

**Background:**

A new web-based food frequency questionnaire (the ASSO–FFQ) was developed within the ASSO Project funded by the Italian Ministry of Health.

**Objective:**

The aim of the present study is to assess the validity of the ASSO–FFQ at food groups, energy, and nutrients level.

**Design and subjects:**

The validation study compared the ASSO–FFQ against a weighted food record (WFR) measuring foods, beverages and supplements intake, compiled during the week following the ASSO–FFQ administration. Ninety-two subjects aged 14–17, recruited from secondary schools in Palermo (Italy), completed the ASSO–FFQ and WFR. The intake of 24 food groups, energy, and 52 nutrients were taken as main outcomes. Tests for paired observations, Spearman and Pearson’s correlation coefficients (cc), kappa statistics and classification in quintiles, Bland–Altman plots and multiple regressions, on untransformed and transformed data were used for the statistical analysis.

**Results:**

High cc (≥0.40) were found for soft drinks, milk, tea/coffee, vegetables, and lactose; fair energy-adjusted cc (0.25–0.40) for water, alcoholic drinks, breakfast cereals, fishery products, savory food, fruit juice, eggs, and 19 nutrients. The subjects classified in the same or adjacent quintile for food groups ranged from 40% (alcoholic drinks) to 100% (dried fruit); for energy and nutrients from 43% (phosphorus, thiamin, niacin) to 77% (lactose). Mean differences were not significant for water, soft drinks, meat, sweets, animal fats, milk and white bread, and vitamin B_12_ and folate. Limits of Agreement were broad for all food groups and nutrients. School, gender, alcohol consumption and between meals mainly affected most food groups’ intake differences. Gender stratification showed females had increased Pearson’s cc for energy and 28 nutrients, such as almost all fats, carbohydrates, vitamins and minerals.

**Conclusions:**

The ASSO–FFQ could be applied in epidemiological studies for the assessment of dietary consumption in adolescents to adequately rank food, energy and nutrient intakes at a group level.

Different tools for the assessment of dietary intake at population level have been developed worldwide, all having strength and limitations (1–3). Since the energy and nutrients intake estimated through these tools are subjected to over- or underestimation, the instrument must be previously validated to recognize the degree of error included in the tool estimation and to adjust epidemiologic measurements. Food frequency questionnaires (FFQ) have been demonstrated to be valid in ranking subjects on a range of food and nutrient intakes (4–8) even though there is an on-going need for the refinement of these existing approaches (9).

The ASSO (Adolescents and Surveillance System for the Obesity prevention) Project funded by the Italian Ministry of Health and involving different national and international partners, aims at developing an innovative web-based system for a standardized collection of data on food consumption, behaviors, and lifestyles in adolescents (10). To this purpose, an ASSO-toolkit was developed within the project (11), including different instruments for the measurement of body composition and fitness, and questionnaires for the collection of lifestyle-related behaviors and food habits. The ASSO–FFQ is one of these tools, designed for the assessment of adolescents’ food consumption. This tool was developed following a systematic literature review (9) and a meta-analysis (Tabacchi G, Filippi AR, Amodio E, Jemni M, Bianco A, Firenze A, Mammina C 2014, unpublished observations) specially performed to investigate the validation of the FFQ in adolescents. Both articles offered indications on the need to develop a new semi-quantitative FFQ that could be valid, reproducible, user-friendly, fast, and provided suggestions on its design and validation. The reproducibility of the ASSO–FFQ was investigated, showing the reliability of this instrument to correctly estimate food groups, energy and nutrients intake in adolescents (Filippi AR, Amodio E, Napoli G, Breda J, Bianco A, Jemni M, Censi A, Mammina C, Tabacchi G. The web-based ASSO-food frequency questionnaire for adolescents: relative and absolute reproducibility assessment. Nutrition Journal, 2014, 13:119. DOI: 10.1186/1475-2891-13-119).

The present study describes the comparative validity of the developed ASSO–FFQ against a 7-day weighted food record (WFR), designed to estimate food groups, energy and nutrients’ intake.

## Methods

### Study design and subjects

A representative sample of 92 boys and girls aged 14–17 was recruited between 2012 and 2013 from three high schools in Palermo (Italy), including one lyceum, one technical, and one professional institute. Students were provided with an informed consent to be signed by their parents and an information sheet explaining the purposes and the methodologies of the ASSO Project. Participants were asked to compile the web-based ASSO–FFQ, and on the same day a hardcopy of a 7-day WFR was distributed to them to be filled in the week following the ASSO–FFQ compilation.

The ASSO Project also involved teachers of the selected schools, who were appropriately trained through a PowerPoint presentation and a specially designed web-based tutorial, realized to well manage materials and tools of the data collection and to carefully assist students during their web compilation.

The ethical committee of the Azienda Ospedaliera Universitaria Policlinico ‘Paolo Giaccone’ in Palermo approved the study protocol (approval code n.9/2011) and all participants provided written informed consent.

### ASSO–FFQ design

The ASSO–FFQ is a web-based semi-quantitative questionnaire included in the ASSO–NutFit software properly developed by the ASSO team.

The NutFit software accessed by students surveyed was in Italian language; also an English version was implemented to make the software accessible for international populations.

It requires on average 20 min to be compiled and has been developed with close ended answers, in order to allow a more direct and simple elaboration of data. It is structured in three sections (foods, beverages and dietary supplements) including 20 major groups: 12 food groups (fruit/vegetables/legumes, cereals/bread/substitutes, pasta/rice/couscous, potatoes, sweets, cheeses/yogurt, fishery products, meat, eggs, fats/oils, savory foods, regional dishes); seven beverages groups (water, soft drinks, juice/milkshakes, milk, tea, coffee, alcoholic drinks), and one dietary supplements group. Each main group is distinguished into different subgroups, for a total of 106 items. The adopted classification considered both raw and cooked vegetables and different cooking techniques for some foods, such as for potatoes.

The analyses were based on data collected between November 2013 and February 2014. The questionnaire asked to indicate the portion size and the frequency of consumption in the previous month. Portion size was assessed through the use of three pictures showing three sizes of the food/beverage (small, medium, large), and when necessary household units were used. Eight possible classes of frequency were proposed: never, 1–2 times/month, once/week, 2–4 times/week, 5–6 times/week, once/day, twice/day, 3–5 times/day.

### WFR design

Reference method for the comparative analysis was a 7-day WFR, properly developed by the ASSO team. It asked to report every food/beverage/supplement consumed during a week, with details on the type of main meal (from breakfast to dinner) and between meals, the time and place, the food/beverage/supplement brand and weighted quantity, and condiments. It was asked to weigh raw foods through the use of a weight scale, and to weigh separately each component of a composed food (e.g. pasta with tomato and parmesan, sandwich with ham and cheese, etc.). Notes were also included to indicate measurement units (mainly g, ml and household units) and other suggestions for a correct compilation of the food record. After data collection, a reviewing process was performed by the ASSO team, in order to identify commonly missing items (oils, butter, etc.) or clearing up illegible terms.

### Data treatment

The Italian tables of nutrient composition of INRAN (12) were used to create a database including the mean values of all nutrients that composed the different foods belonging to main groups. When content values were missing, the food composition databases of USDA (United States Department of Agriculture) (13) were consulted; for brand-named products, the related food labels were retrieved. Standard recipes and serving sizes were used to estimate the nutritional composition of preparations that were not included in the software database. Data from FFQ were then automatically transformed into daily estimated energy and nutrient intakes by the ASSO–NutFit software.

Data from the paper-based WFR were collected and transferred into the software, and, such as those from the FFQ, were automatically transformed into food/beverage/supplement daily frequencies, in order to perform the validation study.

To gather all the foods in groups having a similar nutrient composition, a final number of 24 food/beverage items were considered for the purpose of the validation study: vegetables, fresh fruit, dried fruit, nuts, legumes, breakfast cereals, white bread, bread substitutes, pasta/rice/couscous, potatoes, sweets, cheeses/yogurt, fishery products, meat, eggs, animal fats, oils, savory food, water, soft drinks, fruit juice, milk, tea/coffee, alcoholic drinks.

With regards to the nutrient analysis, a total of 52 nutrient values were considered as outcomes: total fat, SFA, myristic acid, palmitic acid, stearic acid, MUFA, oleic acid, PUFA, linoleic acid, linolenic acid, arachidonic acid, EPA, DHA, TFA, cholesterol, proteins, arginine, cystine, phenylalanine, isoleucine, histidine, leucine, lysine, methionine, tyrosine, threonine, tryptophan, valine, carbohydrates, sugar, fructose, lactose, sucrose, starch, fiber, water, calcium, phosphorus, iron, magnesium, vit A (RAE), thiamin, riboflavin, niacin, vit B_6_, folate, vit B_12_, vit C, vit D, vit E, ethanol, caffeine. Furthermore, the value of energy intake was considered. A total of 15 FFQs and one WFR were excluded from this step of analysis due to implausible energy intakes (>7,000 kcal/day).

### Statistical analyses

Statistical analyses were performed in two phases.

The first phase included food groups’ intake validity analysis. Mean, median, and interquartile ranges of food group intake (measured in g and ml) were estimated both for WFR and for ASSO–FFQ; the intake estimates were not normally distributed, as evinced from the Shapiro–Wilk test. Estimates of untransformed intakes in g and ml were considered in non-parametric methodologies to compare the ranking of individuals by food group: the Wilcoxon signed rank test for difference between paired observations and Spearman rank correlation coefficient (cc) were performed to assess, respectively, the turn-out and the degree of agreement between the rankings of these two methods.

Kappa (k) was used as a measure of agreement (14) between ASSO–FFQ and WFR, and was weighted to take into account the degree of disagreement between the two instruments. The following thresholds established by Landis and Koch (15): ≤0=less than chance agreement; 0.01–0.20=slight agreement; 0.21–0.40=fair agreement; 0.41–0.60=moderate agreement; 0.61–0.80=substantial agreement; 0.81–0.99=almost perfect agreement. The food, energy, and nutrient intakes estimated by the FFQ and the WFR were categorized into quintiles, and the proportion of subjects categorized in the same quintile by both methods, in the same or adjacent quintile and in all the other quintiles (misclassification) was estimated.

Agreement in estimating absolute intakes was assessed by using parametric procedures for which log-(natural) transformed estimates were considered in order to achieve a normal distribution. A small quantity of 1 was added to estimates before making them linear with the log-transformation if participants did not consume some food group. A conservative formulation in case of small sample (*n*<100) was suggested to calculate the Limits of Agreement (LOA), described by Bland and Altman (16), to allow that one method, that is, ASSO–FFQ, can substitute the WFR (17):

$$\bar{d}\pm t_{n-1,0.05}sd\sqrt{\left(1+1/n\right)}$$

where $\bar{d}$ is the mean difference between methods, *sd* is the standard deviation of the difference between methods, t_*n*−1,0.05_ is the value of *t* corresponding to two-sided *p*=0.05 for *n*−1 degrees of freedom and $\sqrt{\left(1+1/n\right)}$ is an adjustment for small sample size.

LOA by food groups were obtained by overlaying the plot of difference versus mean between the two methods. The exponentiated mean difference provided the ratio of intake estimated by ASSO–FFQ relative to the WFR, while the exponentiated LOA ranging between 50 and 200% indicated an acceptable agreement (18).

Student’s *t*-tests for paired samples by food groups were carried out on transformed data to indicate whether, on average, the ASSO–FFQ consistently overestimated or underestimated the WFR. Food groups showing significant difference between ASSO–FFQ and WFR were deeply investigated to identify factors associated with the validity of ASSO–FFQ intake estimates.

Multiple regression analysis was performed with the (transformed) difference of food intakes between the two methods as dependent variable, and personal characteristics, lifestyles and food habits of participants as explanatory variables. When any factor was significantly associated with the differences, it would have indicated that this factor was associated with the validity of the measures.

Personal characteristic, lifestyles, and food habits assessed included: type of school attended (lyceum, professional institute, technical institute); age; gender (male, female); weight status (underweight, normal weight, overweight, obese) estimated according to the body mass index cut-off based on international data (19) (weight and height were directly measured by the properly trained teachers); diagnosed diseases (yes, no); use of dietary supplements (yes, no); slimming regime (yes, no); being sedentary (yes, no) estimated according to adolescents watching TV or using pc/videogames for more than 3 h/day; smoking (yes, no); alcohol consumption (yes, occasionally, no); adequacy of meals (yes, no) estimated on the absence of junk food; between meals (more than one, one, none); organic food consumption (yes, no).

For food groups showing significant dependences of the differences in intake on the mean intake, the multiple regression model included also the mean intake as a predictor of the difference in intake estimates. *R* ^2^ was calculated to quantify the proportion of total variation explained by the explanatory variables all together.

In the second phase, energy and nutrients intakes were considered. The statistical analysis was performed following the same methodology as for food groups, with additional cc on the normalized data that were energy-adjusted weighting for the daily energy intake; a conclusive comparison between raw unadjusted, raw transformed and energy-adjusted (E-adj) transformed Pearson’s cc was showed for all subjects and by gender categories. No regression analysis was performed on energy and nutrient intakes.

All statistical tests were two-sided and a significance level was considered at *p<*0.05. All data were analyzed by using the statistical software STATA/MP 12.1 (StataCorpLP, College Station, TX, USA).

## Results

### Food groups

All 92 subjects aged 14–17, mean age 15.8±1.34 years, compiled the FFQ and WFR were included in the validity analysis for food groups.

Estimated median intakes from ASSO–FFQ were at least twice those estimated from WFR for 15 of the 24 food groups considered (Table 1). Wilcoxon signed rank test assessed significant difference between the medians for 19 food groups: only white bread, sweets, eggs, animal fats, and soft drinks estimated intakes from ASSO–FFQ were not significantly different with respect to the WFR.

**Table 1:** Median, interquartile range, cc, quintiles, weighed kappa, mean difference, and 95% LOA of food intakes

Food groups	Median WFR	Interquartile range WFR	Median FFQ^a^	Interquartile range FFQ	Spearman’s *r* b	% classified in the same quintile	% classified in the same or adjacent quintile	% misclassified	Weighed kappa	Mean difference^c^	95% LOA^d^
Vegetables (g)	46.2	26.9–86.1	127.0***	60.9–234.4	0.48	28	66	34	0.30	0.78***	−2.02–3.58
Fresh fruits (g)	21.4	0.0–93.2	124.6***	35.3–307.1	0.20	25	68	32	0.19	1.88***	−3.68–7.45
Dried fruits (g)	0.0	0.0–0.0	0.0***	0.0–0.4	0.23	29	100	0	0.09	0.23***	−1.02–1.49
Nuts (g)	0.0	0.0–0.0	0.2***	0.0–0.6	0.12	30	53	47	0.04	0.21***	−0.84–1.27
Legumes (g)	0.0	0.0–7.7	16.4***	6.3–41.9	0.19	25	55	45	0.10	1.63***	−1.71–4.97
Breakfast cereals (g)	0.0	0.0–0.0	0.0***	0.0–4.8	0.34	22	41	59	0.33	0.74***	−2.00–3.47
White bread (g)	54.3	26.4–85.2	55.0	22.9–90.0	0.02	19	53	47	0.06	0.02	−3.46–3.51
Bread substitutes (g)	0.0	0.0–13.9	18.1***	7.3–34.8	0.08	22	55	45	0.04	1.44***	−2.39–5.27
Pasta/rice/couscous (g)	55.6	37.9–77.3	124.9***	61.8–202.9	0.00	25	53	47	–0.06	0.47**	−2.80–3.73
Potatoes (g)	26.4	11.4–42.9	55.7***	31.4–95.7	0.10	22	52	48	0.12	1.02***	−2.72–4.77
Sweets (g)	57.0	23.1–93.8	59.1	35.5–146.6	0.25	23	59	41	0.25	0.28	−3.12–3.68
Cheeses/yogurt (g)	18.2	5.4–35.7	38.0***	8.4–119.0	0.16	14	59	41	0.18	0.90***	−3.02–4.82
Fishery products (g)	15.1	0.0–35.3	32.5***	15.2–68.9	0.31	25	65	35	0.24	0.97***	−2.80–4.73
Meats (g)	106.2	83.7–143.0	143.3***	106.5–214.3	0.12	22	52	48	0.10	0.23	−2.31–2.78
Eggs (g)	4.3	0.0–14.3	8.6	2.1–12.9	0.31	18	83	17	0.13	0.52**	−2.71–3.75
Animal fats (g)	0.0	0.0–2.9	0.8	0.2–2.1	0.19	25	74	26	0.15	0.06	−1.93–2.04
Oils (g)	14.7	10.6–20.4	34.5***	22.1–50.5	0.08	27	59	41	0	0.75***	−1.65–3.14
Savory foods (g)	85.7	28.6–126.4	100.0***	63.4–207.3	0.31	23	60	40	0.28	0.68**	−3.11–4.47
Water (ml)	543.6	342.9–919.9	1000.0**	250.0–1500.0	0.25	19	58	42	0.23	–0.26	−6.34–5.83
Soft drinks (ml)	114.3	26.8–214.3	53.6	17.9–223.2	0.46	31	74	26	0.51	0.04	−4.60–4.69
Fruit juice (ml)	0.0	0.0–28.6	14.3***	0.0–85.7	0.32	30	61	39	0.24	1.13***	−3.90–6.16
Milk (ml)	71.4	0.0–214.3	107.1**	8.9–250.0	0.61	53	89	11	0.61	0.47	−4.11–5.04
Tea/coffee (ml)	0.0	0.0–28.9	21.4***	1.8–58.9	0.47	39	88	12	0.46	1.34***	−2.78–5.47
Alcoholic drinks (ml)	0.0	0.0–0.0	26.1***	0.0–105.7	0.37	20	40	60	0.17	2.22***	−1.95–6.40

** *p*<0.01

*** *p*<0.001.

a Medians significantly different (Wilcoxon signed rank test for difference) between paired observations.

b Spearman’s correlation coefficient.

c Significant mean difference between ASSO–FFQ and WFR (Student’s *t*-test for paired observations), estimated on transformed data.

d Lower and upper Limits of Agreement estimated on transformed data through the Bland–Altman method.

Spearman’s cc ranged from 0.00 (pasta/rice/couscous) to 0.61 (milk) (Table 1). Threshold given by Cohen (20) for a high cc (*r*≥0.4) was overtaken by vegetables, soft drinks, milk and tea/coffee, although breakfast cereals, sweets, fishery products, eggs, savory food, water, fruit juice, and alcoholic drinks had a cc≥0.25, a medium value that can be considered acceptable. Lower values were found for the other groups.

The percentage of individuals correctly classified into the same quintile ranged from 14% (cheeses/yogurt) to 53% (milk), while the percentage of correctly or adjacent classified ranged from 40% (alcoholic drinks) to 100% (dried fruit) (Table 1). The weighted kappa values showed moderate/substantial agreement (*k*=0.41–0.80) for soft drinks, milk and tea/coffee; for vegetables, breakfast cereals, savory foods, sweets, fishery products, water and fruit juice a fair agreement (*k*=0.21–0.40) was found; all the other nutrients had slight agreement (*k*=0.01–0.20) except pasta/rice/couscous and oils, which showed lack of agreement (Table 1).

**Fig. 1:**
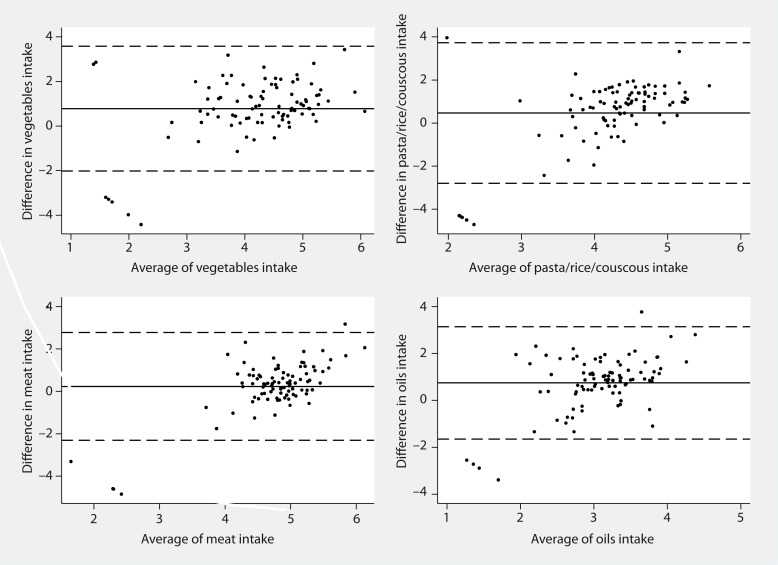
Bland–Altman plots for the comparative validity analysis of vegetables, pasta/rice/couscous, meat, and oils. The solid horizontal lines indicate the mean difference between the two methods and the broken horizontal lines indicate the lower and upper Limits of Agreement (±t_91;__0.025_SDs).

Mean differences estimated through the Bland–Altman method on transformed data showed that there is an overall overestimation of intake from ASSO–FFQ compared to the WFR, and the Student’s *t*-test revealed that this difference is significant for 17 food groups (Table 1). When the exponentiated values of mean differences (mean ratio) were considered, they were higher than 2 for 13 food groups, namely vegetables, fresh fruit, nuts, legumes, breakfast cereals, bread substitutes, potatoes, cheeses/yogurt, fishery products, oils, fruit juice, tea/coffee, alcoholic drinks (data not shown). The LOA were too wide in all food groups, according to the thresholds suggested above; however, all food groups included at least 90% of units within the LOA (Table 1). This is shown for vegetables, pasta/rice/couscous, meat and oils intake in Fig. 1, where the differences obtained by the two methods in each subject are quite well distributed around their mean and they are within the LOA in almost all subjects.

Successively, for the 17 food groups whose mean difference resulted significant a relative validity analysis was performed, for which each food group was included as response variable in a regression model. The multiple regression models revealed two subgroups of the included variables, according to the higher or smaller intake’s differences with the baseline categories (Table 2). The differences were smaller in those adolescents who attended a technical institute, compared to the other types of school, for fresh fruit, bread substitutes, savory food, fruit juice, and alcoholic drinks. Smaller differences were found for female compared to male adolescents in estimated intake of dried fruit, savory food, and alcoholic drinks. Obese adolescents had a mean difference higher than the others for fresh fruit. Subjects who have diseases diagnosed showed a smaller difference for vegetables. Bread substitutes were significantly associated with the adoption of a slimming regime. Smaller differences in legumes’ intake were found for sedentary subjects, whereas smokers showed smaller differences for oils. Alcohol consumers (both regular and occasional) showed higher differences in food intake compared to non-consumers for eggs and tea/coffee. Between meals involved a higher mean difference for fresh fruit, bread substitutes and savory food. Consumption of organic food showed higher differences in fresh fruit estimated intake. Age, use of dietary supplements, and adequacy of meals were found not to be associated with differences for any food group estimated intake.

**Table 2:** Regression models with difference in intake estimates as response and personal characteristics as explanatory variables

		School^a^				Weight status^c^								Alcohol consumption^i^			Between meals^k^				
													
Food groups	Intercept	Lyceum	Technical institute	Age	Gender^b^	Obese	Underweight	Overweight	Diagnosed diseases^d^	Dietary supplements^e^	Slimming regime^f^	Sedentary^g^	Smoking^h^	Occasionally	Yes	Adequacy of meals^j^	More than one	One	Organic food consumption^l^	Mean of FFQ and WFR^m^	*R* ^2^
Vegetables	−1.12	−0.01	−0.81	0.09	−0.26	−0.60	0.74	−0.30	−0.73**	−1.24	−0.04	0.06	−0.37	0.04	0.17	−0.60	0.08	0.19	0.30	0.35*	0.36
Fresh fruits	0.49	−1.23	−2.80**	0.22	0.05	2.49*	0.69	−0.26	−0.76	0.22	−0.31	0.12	−0.97	0.23	0.09	−0.13	0.24	0.88*	0.98**		0.26
Dried fruits	0.49	0.22	−0.08	0.03	−0.47**	0.03	0.02	0.08	−0.59	0.42	0.07	0.19	0.15	−0.30	−0.46	0.13	0.13	−0.51	−0.05	0.69***	0.31
Nuts	−0.05	−0.08	−0.15	0.02	−0.23	0.05	−0.02	−0.19	0.10	−0.54	0.04	0.49	−0.37	−0.28	−0.43	−0.16	0.19	0.34	0.18	0.83***	0.37
Legumes	−1.61	−0.41	−0.89	0.20	−0.33	0.41	1.16	−0.25	−0.97	0.42	−0.51	−1.24*	−0.89	0.33	0.61	−0.14	0.41	−0.09	0.48		0.24
Breakfast cereals	4.68	0.44	0.43	0.29	0.08	0.27	−1.19	−0.03	0.10	−0.18	−0.02	0.16	0.03	−0.10	−0.31	0.06	−0.04	−0.12	0.02	1.14***	0.53
Bread substitutes	−1.31	−0.89	−1.92**	0.30	−0.54	−0.11	−0.16	0.15	0.42	−0.77	−1.28*	0.00	−0.18	0.14	0.19	−0.01	0.76	0.97*	−0.34	−0.76***	0.46
Pasta/rice/couscous	−6.88**	0.58	−0.45	0.17	0.36	0.52	−0.17	0.14	−0.39	0.28	−0.57	−0.36	0.65	−0.01	0.11	0.17	−0.08	0.55	0.33		0.22
Potatoes	2.46	−0.95	−1.14	0.04	−0.01	−1.18	0.24	−0.55	−0.52	−0.40	−0.36	−0.59	0.52	0.23	0.42	−0.11	0.73	0.67	0.40		0.15
Cheeses/yogurt	0.49	0.15	0.07	0.03	−0.02	0.24	0.64	−0.10	0.00	0.11	0.17	−0.05	0.03	−0.11	0.11	0.05	−0.07	0.05	−0.04	0.59*	0.20
Fishery products	1.48	−0.67	−1.12	0.04	−0.21	−1.44	1.86	−0.06	−0.03	0.58	−0.38	−0.42	−1.17	0.42	0.39	−0.13	−0.07	−0.19	0.36		0.19
Eggs	1.23	−0.28	−0.70	0.09	−0.07	−0.23	0.69	−0.19	0.71	0.46	−0.88	0.16	−0.87	1.94**	2.06*	−0.14	−0.15	0.07	−0.36	0.10	0.44
Oils	−2.46	0.26	−0.11	0.03	0.44	−0.11	0.78	−0.05	−0.02	−0.70	−0.03	0.55	−0.92*	−0.48	−0.27	0.08	0.28	0.31	0.15	1.05***	0.49
Savory foods	2.83	−0.21	−1.21*	0.11	−1.14*	−0.58	−0.39	−0.19	−0.45	0.03	0.05	0.64	−0.42	0.65	0.25	−0.14	0.69	1.04*	0.38		0.26
Fruit juice	−6.06	−0.30	−1.79*	0.45	−0.02	−1.03	1.49	−0.86	0.10	−1.33	−0.84	0.21	0.43	0.86	−0.40	−0.05	0.77	1.15	0.96		0.30
Tea/coffee	0.53	−0.63	−0.90	0.03	0.05	0.27	−1.00	0.31	0.67	−0.31	−0.91	−0.11	−0.80	1.43*	1.78*	−0.53	0.32	0.55	0.37		0.22
Alcoholic drinks	−1.80	−1.17	−1.44*	0.27	−1.98**	−0.70	−0.31	−0.76	0.33	0.11	−0.32	0.70	−0.32	−0.02	−1.05	−0.94	0.53	0.37	0.04	−0.20	0.29

* *p*<0.05

** *p*<0.01

*** *p*<0.001.

a Reference category: professional institute.

b Reference category: male adolescents.

c Reference category: normal weight.

d Reference category: no disease.

e Reference category: no supplement.

f Reference category: no slimming regime.

g Reference category: no sedentary lifestyle.

h Reference category: no smoking.

i Reference category: no alcohol consumption.

j Reference category: appropriate meals.

k Reference category: no between meals.

l Reference category: no organic food consumption.

m Included only for multivariable models in which the mean of the intake was significantly associated with the difference in intakes.

No observed variable was found to be associated with the mean difference in food intake for nuts, potatoes, cheeses/yogurt, and fishery products.

The multiple regression models showed that for seven food groups out of the 17 groups, differences were significantly dependent on the mean intake: in particular, for six food groups (vegetables, dried fruit, nuts, breakfast cereals, cheeses/yogurt, and oils), the association was positive, with the differences significantly increasing with the magnitude of intake; for bread substitutes, differences were significantly larger at lower levels of intake (Table 2). *R* ^2^ ranged from as low as 15% (potatoes) up to 53% (breakfast cereals), explaining at least 25% of the difference variability for 11 of the 17 food groups considered.

### Energy and nutrients

Out of the 92 participants, a final number of 76 subjects (65 males and 11 females) were included in the validity study for energy and nutrients, after eliminating all the implausible values related to the high energy intake estimated from the reported food consumptions in the ASSO–FFQ. The mean age was 15.8±1.26 years.

As shown in Table 3, estimated intakes from ASSO–FFQ were at least twice those estimated from WFR for 12 of the 52 nutrients considered: SFA, DHA, TFA, methionine, fructose, lactose, sucrose, starch, niacin, vit C, vit D, and caffeine. Wilcoxon signed rank test revealed significant differences between the ASSO–FFQ and WFR for all nutrients, except for vit B_12_. The percentage of individuals correctly classified into the same quintile ranged from 9% (fructose) to 41% (lactose). The percentage of correctly or adjacently classified ranged from 43% (phosphorus, thiamin, and niacin) to 77% (lactose). Misclassification was higher for phosphorus, thiamin and niacin.

**Table 3:** Median, interquartile range, quintiles, weighed kappa, mean difference, and 95% LOA of energy and nutrients

Energy and nutrients	Median WFR	Interquartile range WFR	Median FFQ^a^	Interquartile range FFQ	% classified in the same quintile	% classified in the same or adjacent quintile	% misclassified	Weighted kappa	Mean difference^b^	95% LOA^c^
Energy (kcal)	1960.14	1658.12–2479.46	3100.39***	2339.35–4307.59	18	51	49	−0.01	0.43*	−0.63–1.50
Total fat (g)	88.18	67.40–107.58	127.58***	94.98–199.31	23	59	41	−0.02	0.40*	−0.77–1.57
SFA (g)	25.70	19.35–34.99	64.55***	43.28–170.67	27	61	39	0.04	1.17*	−1.07–3.42
Myristic acid (g)	0.70	0.47–1.08	1.26***	0.90–1.88	24	54	46	0.01	0.33*	−0.56–1.21
Palmitic acid (g)	10.69	8.33–13.53	17.23***	12.00–25.88	22	49	51	0.00	0.46*	−0.67–1.60
Stearic acid (g)	4.58	3.50–5.54	7.96***	5.90–11.85	27	51	49	0.00	0.50*	−0.57–1.58
MUFA (g)	44.19	30.88–57.41	62.58***	41.12–100.09	20	54	46	−0.02	0.36*	−1.06–1.77
Oleic acid (g)	19.20	12.73–28.25	30.78***	21.13–50.41	20	53	47	0.02	0.50*	−1.00–2.01
PUFA (g)	17.33	9.12–29.50	25.00***	13.81–47.84	22	49	51	−0.07	0.42**	−1.74–2.59
Linoleic acid (g)	13.74	7.32–24.44	21.21***	11.71–41.95	23	50	50	−0.07	0.45**	−1.83–2.73
Linolenic acid (g)	0.84	0.66–1.02	1.08***	0.79–1.71	14	49	51	0.00	0.17*	−0.41–0.75
Arachidonic acid (g)	0.12	0.09–0.16	0.15***	0.10–0.22	28	58	42	0.17	0.05*	−0.15–0.24
EPA (g)	0.05	0.03–0.08	0.10***	0.05–0.18	27	65	35	0.14	0.06*	−0.13–0.24
DHA (g)	0.09	0.06–0.14	0.20***	0.09–0.37	23	61	39	0.15	0.12*	−0.23–0.46
TFA (g)	0.17	0.09–0.36	0.94***	0.55–1.37	28	64	36	0.00	0.46*	−0.26–1.18
Cholesterol (mg)	233.97	192.79–283.39	311.88***	218.84–459.11	27	55	45	0.01	0.31*	−0.96–1.58
Proteins (g)	82.08	72.24–101.40	111.72***	87.75–166.55	24	54	46	−0.07	0.32*	−0.72–1.36
Arginine (g)	3.32	2.89–4.12	5.20***	3.84–7.01	18	55	45	0.03	0.37*	−0.52–1.25
Cystine (g)	0.95	0.77–1.08	1.29***	0.95–1.78	16	47	53	0.02	0.19*	−0.37–0.75
Phenylalanine (g)	2.75	2.30–3.21	4.31***	3.27–5.83	15	46	54	0.02	0.36*	−0.47–1.20
Isoleucine (g)	2.82	2.32–3.34	4.03***	3.04–5.45	19	51	49	0.05	0.28*	−0.54–1.10
Histidine (g)	1.87	1.50–2.16	2.91***	2.24–4.08	18	55	45	0.02	0.37*	−0.43–1.16
Leucine (g)	8.18	4.60–13.62	11.39***	7.49–24.82	16	50	50	−0.04	0.40**	−1.66–2.46
Lysine (g)	7.34	3.81–12.97	11.43***	6.52–23.85	16	50	50	−0.02	0.44*	−1.65–2.53
Methionine (g)	1.42	1.13–1.70	2.86***	2.03–4.02	19	59	41	0.03	0.49*	−0.34–1.32
Tyrosine (g)	2.18	1.80–2.64	3.22***	2.62–4.66	19	54	46	0.06	0.33*	−0.49–1.16
Threonine (g)	2.49	2.02–3.00	3.63***	2.66–5.03	16	54	46	0.04	0.30*	−0.51–1.12
Tryptophan (g)	0.76	0.61–0.89	1.45***	1.01–1.95	24	64	36	0.03	0.34*	−0.29–0.98
Valine (g)	3.22	2.67–3.89	4.74***	3.63–6.33	14	50	50	0.05	0.30*	−0.55–1.14
Carbohydrate (g)	201.32	166.78–274.43	355.54***	264.73–490.95	15	47	53	0.03	0.49*	−0.63–1.61
Sugar (g)	52.80	41.32–73.20	102.77***	74.64–163.10	19	54	46	0.05	0.69*	−0.75–2.12
Fructose (g)	4.94	2.30–6.39	18.61***	8.99–29.37	9	49	51	0.01	1.27*	−1.01–3.55
Lactose (g)	3.64	0.19–10.86	9.34***	2.18–13.06	41	77	23	0.52	0.44*	−1.48–2.36
Sucrose (g)	3.98	2.29–7.51	13.42***	10.21–17.43	19	45	55	−0.02	0.98*	−0.54–2.51
Starch (g)	38.56	28.24–57.94	101.84***	77.20–152.51	26	46	54	−0.03	1.02*	−0.59–2.63
Fiber (g)	23.22	17.86–29.20	30.64***	21.97–45.73	23	49	51	−0.05	0.27*	−0.88–1.42
Water (ml)	888.07	674.94–1117.07	1444.95***	953.47–1864.53	24	59	41	0.07	0.45*	−0.71–1.60
Calcium (mg)	799.95	631.96–1052.12	1055.31**	773.69–1458.74	23	51	49	0.10	0.28*	−0.94–1.49
Phosphorus (mg)	1197.28	1024.73–1445.84	1591.44***	1224.16–2315.66	16	43	57	−0.03	0.28*	−0.71–1.27
Iron (mg)	14.70	12.88–17.96	22.80***	16.50–31.07	18	59	41	0.04	0.35*	−0.74–1.43
Magnesium (mg)	226.18	184.80–284.20	350.35***	246.29–454.13	18	54	46	0.04	0.37*	−0.63–1.38
Vitamin A (RAE)	928.93	610.99–1581.34	742.82**	432.43–1143.71	34	59	41	0.22	−0.34**	−1.97–1.29
Thiamin (mg)	1.30	1.13–1.69	1.75***	1.27–2.50	19	43	57	−0.05	0.21*	−0.68–1.10
Riboflavin (mg)	1.71	1.51–2.12	2.11***	1.49–2.72	27	55	45	0.16	0.12*	−0.65–0.89
Niacin (mg)	23.03	18.30–28.77	136.45**	79.32–245.81	18	43	57	−0.02	1.60*	−0.40–3.61
Vitamin B_6_ (mg)	1.39	1.18–1.68	2.48***	1.76–3.58	26	54	46	−0.02	0.41*	−0.57–1.39
Folate (µg)	380.99	268.78–529.21	441.19*	292.57–638.04	14	49	51	−0.08	0.11	−1.27–1.49
Vitamin B_12_ (µg)	7.37	5.35–10.59	8.17	6.02–11.42	34	66	34	0.19	0.10	−1.27–1.47
Vitamin C (mg)	75.66	52.16–102.24	157.56***	77.79–209.28	27	64	36	0.19	0.59*	−0.77–1.95
Vitamin D (IU)	1.45	1.05–2.17	3.89***	2.43–6.13	16	55	45	0.04	0.69*	−0.68–2.05
Vitamin E (mg)	11.08	8.41–14.59	16.54***	10.96–25.31	24	57	43	−0.01	0.32*	−0.97–1.61
Ethanol (g)	0.00	0.00–0.05	1.83***	0.37–5.38	26	55	45	0.24	0.73*	−1.28–2.74
Caffeine (mg)	0.74	0.00–18.90	19.01***	5.92–34.26	23	59	41	0.07	1.42*	−2.44–5.29

* *p*<0.05

** *p*<0.01

*** *p*<0.001.

a Medians significantly different (Wilcoxon signed rank test for difference) between paired observations.

b Significant mean difference between ASSO–FFQ and WFR (Student’s *t*-test for paired observations), estimated on transformed data.

c Lower and upper Limits of Agreement estimated on transformed data through the Bland–Altman method.

For 21 nutrients, the two methods did not show agreement, with weighted kappa values being null or less than 0; for 29 nutrients a slight agreement was found, with kappa values very close to fair levels for arachidonic acid, EPA, DHA, calcium, riboflavin, vit B12 and vit C; fair values were found for vit A and ethanol, while a moderate kappa value only for lactose.

After log-transformation, mean difference and LOA between ASSO–FFQ and WFR were estimated (Table 3). Student’s *t*-test revealed significant difference between the ASSO–FFQ and the WFR for all nutrients, except for vit B_12_ and folate. Exponentiated mean differences were found to be higher than 2 only for SFA, fructose, sucrose, starch, niacin, ethanol, and caffeine (data not shown). For energy intake, a mean ratio of 1.54 was found out, showing that energy intake estimate provided by the ASSO–FFQ was on average 54% higher than the real value.

**Fig. 2:**
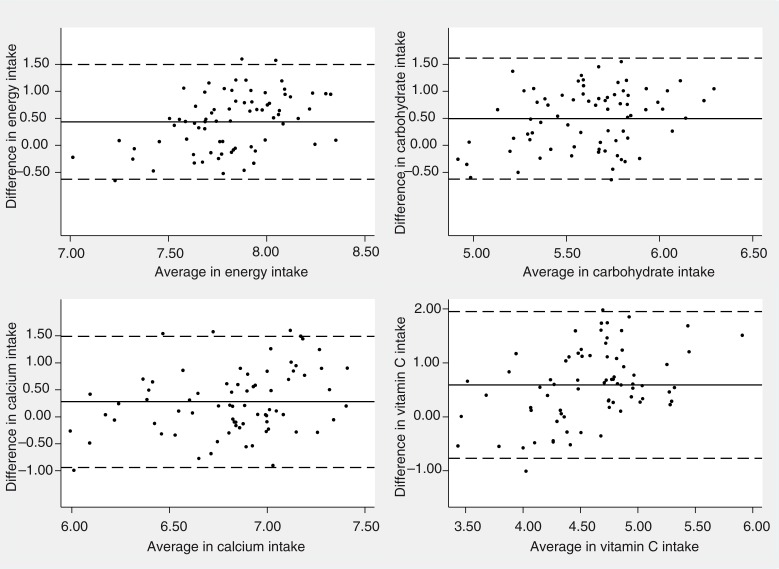
Bland–Altman for the comparative validity analysis of energy, carbohydrate, calcium, and vitamin C. The solid horizontal lines indicate the mean difference between the two methods and the broken horizontal lines indicate the lower and upper Limits of Agreement (±t_91;__0.025_SDs).

Bland–Altman plots for energy and all nutrients showed that differences in intake are distributed quite well around their mean, and they were within the LOA for almost all subjects (only a few units are placed outside), even though the LOA of almost all nutrients were wider than the threshold suggested above. In general, 35 out of 52 nutrients showed at least 90% of units within the LOA and this is reported in Fig. 2 for energy, carbohydrate, calcium, and vit C.

Spearman’s cc and crude and E-adj Pearson’s cc are shown in Table 4. The threshold of 0.4 was overtaken only by lactose, while for all the others nutrients unadjusted Spearman’s and Pearson’s (on transformed data) cc ranged from 0 (several nutrients) to 0.38 (DHA and ethanol); however, most of those cc were not significant. Applying the adjustment for energy intake, cc values became significantly fair (cc≥0.25 and <0.4) for 19 nutrients (Table 4).

**Table 4:** Unadjusted, transformed, and energy-adjusted cc for energy and nutrient intakes, total and split by gender

	Spearman’s *r* a			Pearson’s raw *r* b			Pearson’s Energy-adjusted *r* c		
	
Energy and nutrients	All	Females	Males	All	Females	Males	All	Females	Males
Energy (kcal)	−0.04	0.61	−0.10	0.00	0.51	−0.05	–	–	–
Total fat (g)	−0.03	0.55	−0.09	0.00	0.53	−0.05	0.10	0.19	0.10
SFA (g)	0.11	0.04	0.10	0.16	0.25	0.15	−0.04	−0.10	−0.05
Myristic acid (g)	−0.01	−0.03	−0.02	−0.05	−0.13	−0.05	−0.08	−0.45	−0.02
Palmitic acid (g)	−0.01	0.35	−0.05	0.06	0.50	0.02	0.26*	0.47	0.25
Stearic acid (g)	−0.03	−0.01	−0.05	0.02	0.23	−0.01	0.12	−0.08	0.13
MUFA (g)	0.00	0.43	−0.07	0.01	0.57	−0.06	0.16	0.22	0.15
Oleic acid (g)	0.04	0.10	−0.01	0.12	0.51	0.06	0.04	−0.03	0.03
PUFA (g)	−0.06	0.26	−0.11	−0.04	0.34	−0.10	0.06	0.27	0.04
Linoleic acid (g)	0.13	0.07	0.14	0.06	0.12	0.07	0.00	−0.02	0.01
Linolenic acid (g)	0.00	0.54	−0.07	0.02	0.62	−0.05	0.33**	−0.17	0.38
Arachidonic acid (g)	0.20	0.07	0.26	0.21	0.41	0.23	0.24*	0.63	0.25
EPA (g)	0.35**	0.74	0.31	0.21	0.57	0.19	0.36**	0.73	0.34
DHA (g)	0.38**	0.66	0.34	0.21	0.43	0.20	0.34**	0.60	0.34
TFA (g)	0.11	0.69	0.01	−0.01	0.44	−0.11	0.01	−0.03	0.01
Cholesterol (mg)	0.02	−0.06	0.08	0.09	0.26	0.12	0.13	0.69	0.09
Proteins (g)	−0.11	0.12	−0.13	−0.06	0.10	−0.06	0.17	0.30	0.14
Arginine (g)	0.09	0.13	0.10	0.11	0.19	0.12	0.36**	0.32	0.38
Cystine (g)	0.03	0.09	0.04	0.10	0.00	0.12	0.28*	0.26	0.28
Phenylalanine (g)	0.03	0.12	0.02	0.09	0.03	0.10	0.31**	0.27	0.33
Isoleucine (g)	0.06	0.06	0.06	0.11	0.03	0.12	0.34**	0.35	0.35
Histidine (g)	0.05	−0.06	0.07	0.08	−0.05	0.11	0.32**	0.28	0.34
Leucine (g)	−0.04	0.26	−0.10	−0.02	0.27	−0.07	0.04	0.31	0.01
Lysine (g)	−0.02	0.14	−0.06	−0.01	0.25	−0.05	0.04	0.27	0.02
Methionine (g)	0.10	0.01	0.12	0.11	0.03	0.14	0.30*	0.22	0.33
Tyrosine (g)	0.08	0.01	0.09	0.11	−0.05	0.14	0.34**	0.36	0.34
Threonine (g)	0.07	−0.02	0.09	0.12	0.02	0.13	0.32**	0.27	0.36
Tryptophan (g)	0.09	−0.11	0.12	0.10	−0.05	0.13	0.27*	0.13	0.32
Valine (g)	0.07	0.07	0.06	0.09	0.02	0.11	0.33**	0.37	0.34
Carbohydrate (g)	0.04	0.47	−0.04	0.09	0.47	0.01	0.28*	0.47	0.24
Sugar (g)	0.10	0.36	0.07	0.10	0.45	0.05	0.08	0.30	0.07
Fructose (g)	−0.01	0.36	−0.06	−0.06	0.28	−0.10	−0.08	−0.13	−0.08
Lactose (g)	0.59***	0.66	0.60	0.59***	0.56	0.62	0.54***	0.75	0.52
Sucrose (g)	−0.12	−0.13	−0.11	−0.06	−0.07	−0.06	0.02	−0.51	0.06
Starch (g)	−0.05	0.56	−0.13	−0.04	0.37	−0.09	−0.04	0.23	−0.06
Fiber (g)	−0.05	0.61	−0.16	−0.01	0.52	−0.08	−0.03	0.19	−0.04
Water (ml)	0.13	0.62	0.08	0.15	0.73	0.08	0.12	−0.42	0.17
Calcium (mg)	0.09	−0.15	0.13	0.12	0.17	0.12	0.24*	0.29	0.23
Phosphorus (mg)	−0.06	0.27	−0.11	−0.02	0.33	−0.07	0.28*	0.18	0.29
Iron (mg)	0.05	0.48	−0.01	0.06	0.46	0.04	0.26*	0.50	0.22
Magnesium (mg)	0.07	0.31	0.02	0.11	0.38	0.08	0.22	−0.11	0.26
Vitamin A (RAE)	0.24*	−0.08	0.28	0.35**	−0.08	0.37	0.18	−0.30	0.25
Thiamin (mg)	−0.06	0.47	−0.12	0.03	0.38	−0.03	0.05	0.74	−0.14
Riboflavin (mg)	0.20	0.54	0.15	0.24*	0.55	0.16	0.20	0.37	0.22
Niacin (mg)	−0.04	−0.16	−0.04	0.03	−0.13	0.05	0.14	−0.50	0.15
Vitamin B_6_ (mg)	−0.01	0.34	−0.07	−0.03	0.50	−0.09	0.06	0.64	−0.03
Folate (µg)	0.09	0.38	0.06	0.06	0.28	0.03	0.17	−0.27	0.21
Vitamin B_12_ (µg)	0.20	0.80	0.15	0.17	0.81	0.13	0.12	0.59	0.09
Vitamin C (mg)	0.27*	0.36	0.26	0.36**	0.32	0.37	0.26*	−0.65	0.32
Vitamin D (IU)	0.09	0.03	0.08	0.11	0.40	0.05	0.30*	0.74	0.17
Vitamin E (mg)	0.04	0.53	−0.04	0.00	0.53	−0.08	0.16	0.07	0.17
Ethanol (g)	0.38**	0.30	0.38	0.35**	0.42	0.36	0.18	0.34	0.21
Caffeine (mg)	0.03	0.28	0.02	0.09	0.03	0.12	0.00	0.06	0.02

* *p*<0.05

** *p*<0.01

*** *p*<0.001.

a Spearman’s correlation coefficient; *p*-value assessed by the Wilcoxon signed rank test.

b Raw Pearson’s correlation coefficient on transformed data; *p*-value assessed by the Student’s *t*-test.

c Energy-adjusted Pearson’s correlation coefficient on transformed data; *p*-value assessed by the Student’s *t*-test.

The stratification by gender showed a clear behavior: 21 out of 48 Spearman’s coefficients that first were poor, increased considerably for females (namely energy, total fat, palmitic acid, MUFA, PUFA, linolenic acid, TFA, carbohydrate, sugar, fructose, starch, fiber, water, phosphorus, iron, thiamin, riboflavin, vit B6, folate, vit B12, vit E, and caffeine), and arachidonic acid and vit A cc increased for males (Table 4). Raw Pearson’s cc became fair/high (≥0.25) for energy and 28 nutrients (almost all fats, carbohydrates, vitamins, and minerals); after adjustment for energy intake, 13 Pearson’s cc for females were found to be higher.

## Discussion

In the present study, the comparative validity of a new web-based FFQ in measuring food consumption and energy and nutrients’ intake was evaluated.

Results showed that the tested FFQ is an appropriate tool for ranking adolescents in classes of food groups, even though it is not suitable for measuring the absolute intakes of all food groups. These results are in line with other studies (4–8, 21–24).

Different analytical procedures were used to estimate the validity of the ASSO–FFQ. Based on the comparison between food group medians, the measured intakes of white bread, sweets, eggs, animal fats, and soft drinks were not significantly different between the two methods, thus indicating a high consistency in estimating these foods’ intake by the ASSO–FFQ. The other foods were overestimated, as reported in other studies (25, 26).

Around 50% of the food groups, namely vegetables, breakfast cereals, fishery products, eggs, savory food, water, fruit juice, soft drinks, milk, tea/coffee, and alcoholic drinks, showed fair/high Spearman’s cc values. In this study, vegetable correlation was quite high, and this is not in line with most studies reporting generally low cc values for vegetables (7, 27). Fruit and sweets cc were low, as reported in the study by Vereecken et al. (28).

When the estimation of agreement was performed through the classification into quintiles, results similar to those reported by Deschamps et al. (5) were obtained; on average 63% of individuals correctly classified into the same or adjacent quintile, thus demonstrating that the ASSO–FFQ is able to correctly rank food groups. The foods that best ranked individuals were dried fruits, eggs, fishery products, animal fats, soft drinks, milk, tea/coffee. Misclassification was higher for breakfast cereals and alcoholic drinks, whose assessment through the ASSO–FFQ should be taken into consideration. Breakfast cereals are quite difficult to quantify, as much as the alcoholic drinks, which are often consumed only during the weekend and according to a ‘binge drinking’ approach.

The intake mean differences obtained through the Bland–Altman method for food groups suggested that for 40% of food groups intake estimates provided by ASSO–FFQ were less than twice those estimated from the WFR, a value that we consider acceptable. However, as revealed by the wide LOA obtained, the ASSO–FFQ was not able to correctly estimate absolute food intakes, and this is in line with different validation studies of other FFQs (7, 8). Nonetheless, a good distribution of values around the mean difference was found for most of the food items.

Type of school, gender, alcohol consumption, and between meals were significant explanatory variables of the intake differences between FFQ and WFR, even though weight, diseases, slimming regime, sedentary behavior, smoking, and organic food consumption can influence the validity for some food group. These findings are in line with those reported by others (7, 25, 29) and confirm different levels of validity depending on personal characteristics, lifestyles and food habits of participants. Moreover, differences between the WFR and FFQ were not constant across levels of intake, but were dependent on the magnitude of intake for seven food groups. The models for vegetables, fresh fruits, dried fruits, nuts, breakfast cereals, bread substitutes, eggs, oils, savory food, fruit juice and alcoholic drinks explained at least 25% of the variation in difference between ASSO–FFQ and WFR. The multivariable modeling suggests that ASSO–FFQ is not able to correctly quantify in particular the absolute intake of nuts, cheeses/yogurt, fishery products, and potatoes, and this may be explained by different reasons. As nuts are not a frequently consumed food, adolescents probably did not eat nuts in the week they compiled the food record. For cheeses/yogurt, the need to improve the photographs for the portion size estimation could be discussed. Consumption of fishery products could be affected by the presence of seasonality: lower intake levels reported in the WFR could be explained by a lack of fish availability on the market, due to adverse meteorological conditions. Finally, potato intake estimates were quantified in detail within the ASSO–FFQ, since they were separated according to the different types of cooking (fried, fried in the package, boiled or baked, mashed and potato dumplings) and it is a frequently consumed food; so the disagreement might depend on the photographs used to quantify the portion, which in this case should be modified, or on other factors not accounted in this validation study.

With regards to the nutrients, the authors conclude that the ASSO–FFQ overestimates nutrients intake and is not appropriate to assess absolute intake of most nutrients; however, the FFQ ranks individuals with reasonable accuracy. These findings are in line with different previous studies (4, 5, 8, 24, 30).

The agreement in quintile categorization was similar to that observed by Deschamps et al. (5) and Hong et al. (30). On average, 54% of individuals were correctly classified into the same or adjacent quintile, thus demonstrating that ASSO–FFQ is able to ranking subjects on a range of nutrient intakes. Presenting data categorized in quintiles provides compact information concerning the capacity of both methods to allocate individuals according to dietary intake distribution, and it is considered more adequate than the correlation coefficient, which merely produces information concerning the possible relations between the variables estimated by both methods (6).

The mean agreement and the LOA between the tested FFQ and the WFR obtained according to the Bland–Altman procedures were not acceptable for SFA, fructose, sucrose, starch, niacin, ethanol, and caffeine, since their overestimation was more than double; this is in line with the study from Lietz et al. (26). Mean agreement was good for vit B_12_ that showed no significant difference between the two methods, and was acceptable for all the other nutrients. Even though the Bland–Altman LOA resulted quite wide, the differences in the intake were well distributed around their mean and more than half nutrients had at least 90% of units within the LOA, according to some authors (25, 31).

When Spearman’s cc were considered, values were found to be low/very low for energy and most nutrients, even though they were not significant; with concern to energy, the very low correlation found was in contrast with most authors that reported values >0.20 (26, 32, 33). Only EPA, DHA, lactose, vit C, and ethanol had significant cc>0.25. Although comparison with other studies is difficult due to methodological differences, our findings are in line with other studies reporting low cc for different nutrients (26, 34–37), but disagree with the other validation studies reporting in general fair/high cc (6, 25, 38). After energy adjustment, correlations increased and could be retained fair/high for about half nutrients, such as different subgroups of fats, almost all amino acids, lactose, phosphorus, iron, vit C and vit D. The energy adjustment’s procedure allowed us to obtain more precise and higher estimates of the correlation coefficient, as reported also in other validation studies (26, 30, 33). These higher correlations may be explained by a decrease in correlated measurement error for total energy and nutrients that exceeded the reduction in between-person variations for nutrient intake as a consequence of controlling for total energy intake (33). The decreased correlation coefficients reported after energy adjustment by other authors (6, 39) could be explained if considering that subjects may not have reported foods rich in nutrients such as fat in the same way during assessment for both dietary intake methods (30). Instead, for total fat, SFA (excepted palmitic acid), MUFA, PUFA (with the exception of linolenic acid, EPA and DHA), TFA, cholesterol, proteins, sugar, fructose, sucrose, starch, fiber, water, calcium, magnesium, and most vitamins, the ASSO–FFQ validity seems to be low even after adjustment for energy, thus it is not accurate in determining the magnitude of intake for these nutrients. These results are discouraging if gender was ignored; in fact, when the stratification by gender was assessed, the correlation increased significantly in females for most nutrients, since females should report intakes more correctly, as already suggested by different authors (29–31, 40). Our results may then be explained by the low number of females recruited in the study compared to males, which could have affected the validity of the FFQ, also considering that the ASSO–FFQ did not perform as well for the females as it did for the males in adequately classifying individuals according to their nutrient intakes. This could indeed be considered one limitation of our study.

When comparison of food and nutrients intake validity through cc was performed, intakes of both milk and lactose (which is contained almost exclusively in milk) showed good correlation; the same happened with EPA and DHA, principally contained in fishery products, which is hypothesized to be affected by seasonal conditions; and with SFA comprised in animal fats, milk, eggs.

In summary, significant correlations were observed for food groups, and significant cc after energy adjustment and after stratification by gender were observed for energy and nutrients, but when assessing the between-method differences using the Bland–Altman analysis, the LOA were quite broad, which is unacceptable for dietary assessment purposes. Thus, ASSO–FFQ is not able to assess absolute intake of foods, energy and nutrients, and the agreement with the WFR estimated by the kappa statistics was very poor for nutrient intakes; however, as its ability to classify individuals into quintiles was good, it could be considered a valid tool for correctly ranking subjects in classes of food, energy and nutrient intakes.

A WFR was adopted as reference method because it does not depend on the recall and it has the potential for allowing direct measurement of food quantities (41); nonetheless, WFR may have the disadvantage of recalling inadequately measure of foods episodically consumed (42), that could have affected the intake report, such as the case for nuts.

As underreporting is the main bias that interferes with the food intake data obtained from food records (43–45), and overreporting is usual in FFQ (4, 24), possible underreporting in the food records and overreporting in the FFQ might have had a synergic effect in influencing the results observed in this study, for example, by increasing the intake mean differences between the two methods. As stated by Vereecken et al. (8), over- and underestimation are important issues and the current findings suggest to increase the cut-off values of the recommended intakes or to apply a correction factor to decrease the reported intake of the nutrients too overestimated (such as fiber, vitamin C, calcium, and iron), and to increase the percentage of energy from fat before being evaluated for feedback.

Twenty-four food groups, energy, and 52 nutrients were analyzed in multiple ways; this could have introduced multiple testing bias and should be taken into account in further studies.

Another aspect is that adolescents have difficulties in recalling what food they have eaten (36), for example, food eaten away from home (46); they can have limited knowledge of food and food preparation or problems in the perception and quantification of the portion size (46). According to Goran (47), adolescents tend to have better recall of the preferred foods, and to forget or underestimate those items that they do not like. Furthermore, the composite dishes (e.g. pasta/rice/couscous with meat) included in the FFQ, by which dishes were classified as the whole mixture or on the basis of its primary ingredient, could play a role (48). According to these considerations, the presence of systematic errors cannot be excluded.

## Conclusions

Findings suggest that the ASSO–FFQ could be applied in epidemiological studies for the assessment of dietary consumption in adolescents to adequately rank food, energy, and nutrient intakes at a group level. The presence of a section where regional/local foods may be selected makes it suitable for use in other regions for the comparison of food and nutrient consumptions. Moreover, it is also developed in the English language and could be easily translated into other languages, with the potential to be applied in other countries after adaptation to the local culture and food habits.
